# The league of extraordinary generalists: a qualitative study of professional identity and perceptions of role of GPs working on a national after hours helpline in Australia

**DOI:** 10.1186/s12913-016-1387-5

**Published:** 2016-04-22

**Authors:** Rosemary McKenzie, Michelle Williamson

**Affiliations:** Centre for Health Policy, Melbourne School of Population and Global Health, The University of Melbourne, Victoria, 3010 Australia

**Keywords:** Australia, Telephone triage, GP identity, Generalists after hours care

## Abstract

**Background:**

Telephone triage and advice services (TTAS) have become commonplace in western health care systems particularly as an aid to patient access and demand management in the after hours period. In 2011 an after hours general practitioner (GP) helpline was established as a supplementary service to existing 24-h nurse-TTAS in Australia. Callers to the service in the after hours period who are triaged by a nurse as needing to see a GP immediately or within 24 h may speak with a GP on the line to obtain further assessment and advice.

While much research has been undertaken on the roles of nurses in TTAS and the professional identities and attitudes to new technology of community-based GPs, little is known of the perceptions of role and identity of GPs providing after hours advice on primary care helplines. This qualitative study explored the perceptions of professional identity and role, motivations and contributions to the health system of GPs employed on the Australian *afterhours GP helpline* in 2011–2013.

**Methods:**

The study took a phenomenographic approach seeking to understand the essence of being a telephone GP, probing professional identity while also exploring role tensions. Twelve GPs, or 15 % of the helpline GP workforce participated in the qualitative study.

**Results:**

The GPs experienced both personal and professional benefits and believed they were strengthening patient care and the Australian health system. However the role required a re-alignment of practice that challenged professional autonomy, the doctor-patient relationship and commitment to continuity of care. Some GPs made this role realignment more readily than others and were well suited to the helpline role. There was a strong collegial bond amongst the helpline GPs which facilitated the maintenance of professional autonomy.

**Conclusions:**

Telephone GP assessment and advice does not demonstrate the same breadth as face-to-face practice and provides little opportunity for continuity of care, but this has not prevented those performing the role from identifying as a new form of generalist. The establishment of an after hours GP helpline in Australia has seen the emergence of a new generalist primary care identity as telehealth innovators.

**Electronic supplementary material:**

The online version of this article (doi:10.1186/s12913-016-1387-5) contains supplementary material, which is available to authorized users.

## Background

Telephone triage and advice services (TTAS) have become commonplace in western health care systems, both as an aid to patient access and for demand management in the after hours period [1–3]. Many such services are staffed by nurses [4–6] with fewer examples of TTAS providing direct access to general practitioner (GP) telephone advice [2].

In 2011 as part of national primary healthcare reforms, an after hours GP helpline was established as a supplementary service to existing 24-h nurse-staffed TTAS in Australian states and territories. Callers to the nurse triage service in the after hours period (6 pm to 8 am weekdays and 1 pm Saturday to Monday morning, plus 24 h on public holidays) who are triaged by a nurse as needing to see a GP immediately or within 24 h can be transferred to a GP on the line to receive assessment of urgency and advice on the service or care that the patient requires. It was not established as a diagnostic service and the GPs do not prescribe or order tests. There is no contact between the triage nurse and GP during or after transfer of the call and most nurses and GPs on the service work from home using a shared IT platform. The triage nurse uses a computerised clinical decision tree to reach a disposition. The GP has more clinical freedom and after confirming essential details, conducts the telephone encounter broadly as he or she sees fit, without reliance on computerised protocols. Online decision-support resources are available and the GP may “instant message” other GPs working on the helpline at the time for a second opinion. The GPs receive, on transfer of the call, an electronic patient encounter record that includes the caller’s telephone number should a call back be required. The record is augmented by the GPs notes. The caller is advised that their regular GP may request a copy of the encounter record from the service. During the study period the encounter record was not routinely provided to the caller’s GP. Callers did not have direct access to the call encounter record during the study period.

The changing professional identity of family or general practitioners has been extensively studied, with particular interest in the identity tensions between generalist practice and the expanding territories of specialisation [7–10]; impact on identity of changing professional boundaries in healthcare [11–15]; the rise of evidence-based medicine [16, 17] and the emergence of new business and management models in general practice [18]. New models of primary care may also shape the skill set and role played by the medical generalist [19].

The attitudes of community-based practitioners to new models of out of hours care [20–23] and the use of telephone and other technologies in health care [24–27] have also been extensively examined. Freidson [28, 29] first explored the notion of professional autonomy as a key feature of the medical profession’s identity, arguing that medical collegiality and group bonding were an expression of a shared belief in the special expertise held autonomously by the profession. However little is known of the sense of professional identity or perceptions of role of GPs who provide primary care assessment as part of a TTAS. This qualitative study explored the perceptions of professional identity and role of GPs employed in the first cohort of doctors working on the Australian afterhours GP helpline (AGPH). It was undertaken as part of a larger mixed method evaluation of the AGPH in its first two years of operation, 2011–2013.

## Methods

### Conceptual framework

The study took a phenomenographic approach seeking to understand the essence of the experience of being a telephone GP, exploring the range of individual perceptions, identifying common themes while also probing role tensions and differences in professional self image [30, 31].

### Setting and sample

The AGPH employed approximately 80 GPs in the study period who worked from their homes using a computerised telephony system to receive calls referred to them by a telephone triage nurse. All GPs received a written invitation to participate in an in-depth semi-structured interview. Procedures were in place to protect the anonymity and confidentiality of helpline personnel and therefore the invitation was circulated electronically by the helpline operator, but respondents indicated their willingness to participate by contacting the researchers’ email address. This protocol ensured that the employer of the GPs did not know the identity of those participating in the study and the evaluator did not have access to names and contact details of the GP workforce of the helpline prior to consent to participate being given. All respondents received information about the study and provided written consent to participate. Ages and residential location of the participating GPs were not sought to protect the privacy of participants. Ethical approval was received from the University of Melbourne’s Human Ethics Research Committee. Twelve GPs agreed to participate in the study representing 15 % of the helpline’s GP workforce. Fifteen GPs expressed an interest in participating but two parties did not respond to further contact and in one case the respondent did not proceed with the interview because it was considered too lengthy. Ten GPs were interviewed by telephone and two GPs asked to respond in writing to the semi-structured interview schedule, providing free text answers and additional reflections on the role. The interview schedule is found in Additional file 1. The first author (RM) interviewed eight respondents and liaised by email with those responding in writing and the second author (MW) interviewed two respondents. Interviews took between 45 min and one hour and 15 min.

### Analysis

Telephone interviews were digitally recorded and transcribed and the interviewers in addition took extensive notes while conducting the interviews. Analysis of the transcripts used the inductive constant comparison method [32–34] with close review of the transcripts and recordings to identify emerging concepts to which a code or theme name was assigned. Ongoing comparison, development and refinement of the codes was undertaken as further interview material was examined, with the principal analyst (RM) discussing and checking concepts and codes with the co-researcher (MW).

## Results

### GP profile and employment on the helpline

Of the 12 GPs who participated in the study seven were female and five were male. The majority of respondents had worked with the helpline since its inception in July 2011 (for a period of 20–22 months depending on the time of interview). All had worked in general practice for at least 5 years, and up to several decades for a small number of respondents (*n* = 3). Eleven of the 12 continued to work in community general practice or medical settings part time, including in rural hospital emergency departments. Only one respondent worked full time on the helpline. Most respondents reported working five or more five-hour shifts per fortnight on the helpline.

### Value of the role to self, patient and health system

GPs conceptualised the role in terms of personal and professional value with the latter encompassing both patient and health system level benefits.

Personal value - work-life balance, financial benefits.

All GPs interviewed enjoyed the lifestyle benefits of a home-based role with flexible, albeit unsociable hours. A number of respondents had young children or other carer responsibilities that the helpline role enabled them to attend to; others enjoyed leading a mixed professional life with some regular clinic or other medical work combined with helpline shifts. GPs on the helpline appreciated what they saw as relatively generous financial remuneration and acknowledged that this provided support for other pursuits, including professional business interests that would otherwise not be possible.

“Personally I like the flexibility, the ability to swap shifts, no commuting, no needing to get dressed for work, working from home” (GP 11 - written response).

“I love working at home and most of us like the dough (sic) [money] too” (GP8).

Professional value - “First responders” to urgent needs

GPs saw their role as a frontline service helping to meet urgent needs of patients and determining appropriate care. One GP described this notion thus:

“We are getting people to care or treatment in time when they need it” (GP8).

Respondents felt that they performed a role that was highly valued by consumers.

“Patients are so happy to talk to us … it’s very rewarding to get such positive feedback as we don't always get this in private practice” (GP 7).

Most respondents saw a number of benefits for patients. These included reassurance and emotional support, education on health conditions and self-care, and provision of a plan when the patient hasn’t known what to do next.

This latter point was well captured by a respondent (GP9) who said:

“We help people to make a plan before they engage with a face to face service. To make the decision to go, it’s a big decision, they don’t want to waste people’s time …we help them decide on a course of action. …The key thing is the immediate guidance – it’s making a world of difference to the patient.”

Another emphasised the telephone GP’s education role:

“I provide more than just advice on the presenting problem … I do a lot of education and help people use services appropriately” GP5.

The capacity to help isolated or high need groups such as older people or parents of infants was mentioned by several respondents:

“There are so many isolated older people – it’s money well spent” (GP3).

Professional value - strengthening the health system.

Helpline GPs had a number of insights into the benefits of the service at a health system level, which included providing better access to after hours care for rural and remote communities and isolated populations such as the elderly and directing people to appropriate after hours services. Several GPs mentioned their belief that they help to avoid health costs by giving patients appropriate advice on what to do next in terms of self-care or waiting to see a GP in regular business hours.

“It has the potential to provide health systems savings. It can direct people away from expensive services that are not necessary and can get people to treatment before they become acute” (GP10).

### Value added by GP to nurse triage and advice

Interviewees had strong views that the addition of a GP to the *healthdirect* nurse line added significant value. There were a number of commonly identified additional benefits. GPs believed their clinical advice is seen as more authoritative by the patient and that the GP can provide more in-depth and directly relevant assessment of the patient’s condition than the nurse protocol driven system allows. One GP’s view typified the comments of the majority.

“We bring more clinical knowledge and skill than a nurse can bring to it” (GP4).

Being able to explain and advise on medication issues was considered important by the GPs. One respondent suggested that GPs were better at handling patient emotions than nurses were able to in their triage role.

“We are helping to manage the person’s feelings about it. Managing the patient’s anxiety and stress level … we offer more than an automated assessment. We can work on the relationship to help them comply with what you want them to do” (GP9).

### Role realignment - diagnose or safely dispose?

The GPs were asked about the characteristics of their telephone role. The majority of GPs felt that their primary role was management of presenting symptoms and appropriate referral to the next point of care. Common across all respondents was the view that this was a very different role from face-to-face clinical practice.

As one GP explained “to be able to do this job you need to understand the role; it’s mainly an advisory and symptom management service” (GP8), while another said, “I had to let go of the notion that I had to, umm, solve the problem. Early on I had to align my expectations of what it meant to be a doctor with the new [helpline] role” (GP7).

A commitment to patient safety was frequently identified as a cornerstone of the role, and was given a higher order importance than clinical accuracy:

“I compensate for not seeing the patient by being extra careful. I’m not here to be right, I’m here to be safe, so I might send five people to ED ‘cos I don't want to miss one case of meningitis” (GP2).

“My job’s not to say what’s wrong, [but] to judge seriousness and what the patient should do next” (GP5).

Most GPs expressed confidence in the safety of the service. They noted that their training emphasised safety at all costs. Many of the GPs used terms such as “risk averse,” “cautious” and “very experienced” to describe themselves and their fellow telephone GPs. Each GP explained to the interviewer the standard “safety net” advice given with each call - to call back if concerned and to seek immediate medical assistance if the condition worsens. However most noted that safety also relied on adequate clinical resources (online guidelines and access to other helpline GPs through instant messaging) and reasonable service performance expectations from management.

### Role limitations and tensions

The majority of respondents identified limitations to the role and more than half (seven respondents) articulated role tensions that were affecting their professional satisfaction.

Most respondents said it was important to have another role in face-to-face practice to ensure that clinical skills were maintained. When probed on this it was found that the GPs thought most helpline calls were straightforward and readily disposed to an appropriate point of care without requiring high-level clinical skills. One GP said “It can be boring and a bit frustrating if you just do after hours telephone work” (GP 7), while another said “it is easy to de-skill if you’re on the phone for 35 h per week” (GP 8). Another commented that she felt the role was limited “because I can’t diagnose and I can’t prescribe” (GP5).

Seven respondents expressed concerns about what they saw as competing pressures to perform in a “call centre” environment with target average handling times (AHTs) compared with their own professional standards of patient care. More female (5) than male (2) GPs articulated this tension.

“Feeling like [management] are a helicopter monitoring us, hovering to try and get us to decrease call times, and to increase the number of patients we speak to. At any time I feel that it is becoming unsafe (pressures from above) I will have no hesitation in leaving.” (GP11 - written response).

“I feel a constant tension in my work. I take time to listen to patients but my average handling times are higher and this doesn’t go down well” (GP3).

### Lack of continuity of care

A smaller number of GPs (*n* = 4) spoke about the loss of relationships with patients and the lack of information about the caller over time, both in terms of the outcome of the health issue and the broader patient history and context that may not be provided in a helpline call.

“I have no ongoing relationship with the patients and I don’t know whether my advice has been taken or has been successful. I would like to see a system whereby interesting cases can be followed up, purely for us to know what happened” (GP12 - written response).

“It is challenging if you can’t resolve the uncertainty – sometimes a patient seems very anxious when the symptoms don't seem to justify urgent care and you wonder what else is happening” (GP 6).

In contrast however three GPs expressed a sense of relief in relinquishing responsibility for ongoing care.

“I like the short interactions – and not having to follow up on every single person. We add value, but without taking on the whole case “(GP9).

### Misunderstood and resented – perceptions of peer responses to their role

Most respondents believed that their community-based medical peers saw the helpline service and the GPs working on it in a poor light. A common view among the helpline GPs was that the broader medical community did not view their telephone role as real clinical work and that they were diverting money from GPs and health services where more resources were needed.

“The medical community look down on the helpline. They think we are just sitting at home sending people to hospital” (GP5).

“Most rural GPs see it as a waste of money…millions of dollars going to a phone line that tells people to go to hospital when they are struggling to deliver care” (GP8).

“There is a very poor understanding of what we do. They think we are taking money from home visits and after hours clinics” (GP 7).

One GP felt embarrassed to tell her peers that she worked on the helpline “because I feel they will look on me as a lesser calibre doctor” (GP11- written response).

Several noted that it was the lack of understanding and knowledge of how the service works that led to these negative perceptions.

“Most GPs don't have a clue how it works. When they understand our role better more of their patients will use it” (GP6).

### Comrades and extraordinary generalists

Despite the home-based nature of the role, a strong collegial network has developed between GPs working on the helpline. At a practical level the collegial network has been fostered by the internal instant messaging system. But GPs also expressed a perception of being pioneers together in a new world of practice. As one GP described the network of peers “It’s different and new. No one has done it before. You are working as part of a team – even though you are by yourself, you are with other doctors” (GP10). This view was confirmed by others:

“It feels like being part of a team even though we are working alone” (GP 2)

“This is the most social job I have…there’s great camaraderie with the other doctors” (GP3).

One GP reported that the GPs had created their own email list to keep in touch when not working. The email group was dubbed “The League of Extraordinary Generalists” taking its name from the 1999 comic book series *The League of Extraordinary Gentlemen*, and the 2002 film of the same name, set in a fictitious, technologically advanced universe, peopled with literary and cinematic heroes.^1^ While clearly a tongue-in-cheek adaptation, the name reveals a shared identity amongst the telephone GPs.

Some respondents expressed a view that their professional skills and knowledge had developed being a helpline GP. “It has made me a better doctor,” one GP (GP7) remarked, while another said “I am learning to think more clearly and objectively about my decisions” (GP11, written response). A third observed “It has helped me think more laterally about what the problem might be” (GP6).

Peer learning was identified as another contributor to improved clinical knowledge.

“It's a really terrific group of doctors with really varied experiences - ED, anaesthesia, rural practice - so there is so much learning from each other” (GP 5).

## Discussion

GPs working on a national after hours helpline perceive great value in their role but experience a number of role tensions and challenges to their professional identity. Three main sources of tension are apparent.

The first is the call centre business model and the associated efficiency constraints, which make the telephone GPs feel pressured to adhere to target times in consultations. This is a pressure that challenges the GPs’ perception of their professional autonomy. Some helpline GPs feel this pressure more keenly than others and express less satisfaction with their role as a result. Others are accepting of the constraints and feel comfortable working within the performance framework of the service. These telephone GPs still conceive of themselves as being autonomous, safe medical practitioners within this new scope of practice, and indeed enjoy the nature of the work. However others are ambivalent and find the business model challenges their core sense of professional self. McDonald and colleagues [18] examined GP attitudes and identity following the implementation of new enterprise models in the UK and found similar ambivalence. Most GPs in the study found a new business model a motivating opportunity to extend or change their practice, while others felt the enterprise model and associated targets either challenged or were extraneous to their autonomous mode of patient-centred practice. Professional autonomy has long been seen as a fundamental plank of medical identity [16, 17, 28, 29]. It would seem that some telephone GPs can adjust their mode of practice without a sense of compromised professional freedom while others find the role impinges on their sense of autonomous professional self.

The second source of tension derives from the essential nature of a patient-initiated, population-based helpline. Adapting to a telephone role that involves only a brief encounter with an unseen, unknown patient who may reside in any part of the nation requires an adjustment not only to the way in which the GP performs his/her role but also to the way in which he/she perceives their professional identity. The doctor-patient relationship is another cornerstone of the general practitioner identity, with an expectation that the GP comes to know the patient and provides care and support over time within the context of the patient’s history [14]. Continuity of care is thought to be a fundamental part of the modern doctor-patient relationship in primary care [10]. The realignment of role, from diagnosis of the patient’s problem and ongoing care, to safe disposal of the patient to another service or self care moves away from these fundamental elements of professional identity. Relinquishing the centrality of the doctor-patient relationship and continuity of care is key to the role realignment required by the telephone GP. Those GPs who readily make this realignment appear to be well suited to the helpline role, while those who miss continuity of care feel greater professional tension and may be less likely to stay in the role.

A third source of tension is the way in which the helpline GP role is perceived by peers in the broader medical community. It appears that the role of the telephone based GP has been either misunderstood or not fully understood by other GPs. The minimal contact between the AGPH and community GPs has possibly exacerbated the lack of understanding and led to some perceived disparagement of their professional worth. Haddow and colleagues [23] found that there was considerable resentment of the inaugural NHS 24 nurse-led telephone service amongst GPs working in after hours cooperatives because of the threat the service represented to their professional autonomy. In the United States, where various forms of telehealth are now well established it has been found that many doctors continue to resist the changes associated with increased availability of telehealth systems [26]. Similarly research in the United Kingdom has found many primary care practitioners are suspicious of care that occurs outside a face-to-face consultation [25, 27]. Resistance to change amongst community GPs may be an underlying factor, however our findings suggest the negative perceptions of telephone GPs held by community GPs are linked to perceived diversion of funds and apparent lack of assistance to community GPs in reducing their workload. In addition a patient’s phone encounter records were made available only at the request of the patient’s regular GP, putting the responsibility on the community GP to prioritise continuity of care for the patient. While negative community GP attitudes appears to have strengthened the sense of collegial solidarity of the telephone GPs, the understanding gap could be overcome by the service operator more actively engaging with the medical community and supporting continuity of care by routine provision of telephone encounter records to the patient’s regular GP.

Despite these tensions, the telephone GPs saw benefits for patients and the health system in the service they provide and they articulated a strong sense of professional identity as capable generalists working in a new field. Although the role was described as more limited in professional challenges and range than face-face to clinical work, the telephone GPs identified new skills gained that had not been developed in face-to-face practice. They also identified themselves as innovators strengthening the national health system by providing a new, needed primary care service which distinguished them professionally from other community-based generalists. This sense of otherness or difference can be seen as a rhetorical means of establishing the boundaries of this particular role [12] from both community-based GPs and the nurses who initially triage callers to the helpline. The group identification as “extraordinary generalists” is a proclamation of the telephone GPs’ belief in their competence and high professional standards in a new practice model. The collegiality that characterises this league of generalists is a further demonstration of their belief that the group, collectively and individually, are highly skilled autonomous professionals, just as Freidson [28, 29] has previously observed of medical collegiality.

Wachter and Goldman [10] suggest that medical generalism is characterised by comprehensiveness and continuity of care. More recent definitions present medical generalism as a philosophy of practice and a particular approach to healthcare that involves a way of thinking and acting that is distinctive to the generalist practitioner and is not confined to a single setting [19]. Telephone GP assessment may not demonstrate the same breadth as face-to-face practice and provides no opportunity for continuity of care, but this has not prevented those performing the role from identifying as a new generalist with a particular approach to delivery of healthcare – the lateral thinking, adaptable telephone GP.

### Limitations

The principal limitation of the study is sample size. The sample is relatively small and may not necessarily be representative of all GPs working on the helpline. We were restricted in our access to helpline GPs and information about the profile of the workforce as the operator is a commercial telehealth provider contracted to the Australian government with commercial in confidence provisions in place. Limited access meant that there was no opportunity to pilot the interview questions or check findings. However the long, semi-structured interview format led to the interviewers gaining an understanding of the characteristics of the respondents and we believe that in the circumstances, it was a diverse and reasonably representative sample, with a mix of age, professional experience, geographic location and gender.

## Conclusions

This study has provided insights into the role and perceptions of identity of GPs working on an after hours telephone helpline. While there are role tensions and varying degrees of professional satisfaction amongst GPs working on the helpline, there is a shared understanding of the role as different, innovative and value-adding to the health system, over and above what might be offered by nurse-led TTAS. The occupation of nurse in telephone triage and advice services has been identified as a new nursing role. This qualitative study suggests that the establishment of an after hours GP helpline in Australia has provided the platform for an emerging primary care generalist role as a telephone-based GP with a distinct professional identity. Further research is needed to understand the development of this emerging role over time and the relationship of the role to other professions involved in delivery of primary care in the community.

### Ethics (and consent to participate)

The study of GP providers of the after hours GP helpline received approval from The University of Melbourne Human Research Ethics Committee, Approval number 1339934.1. All interviewees gave informed consent to participate in the study.

## Availability of data and materials

Ethics approval for the study required that the audiotapes and transcription of the interviews be kept in a locked file accessible only by the authors. We have supplied the interview schedule as an additional supporting file.

## Additional file

Additional file 1: The interview schedule used to guide discussion with participants has been supplied. (DOCX 98 kb)

## Abbreviations

AGPH: after hours GP helpline
GP: general practitioner
TTAS: telephone triage and advice service

## Footnotes

1 For more information on The League of Extraordinary Gentlemen see http://dc.wikia.com/wiki/League_of_Extraordinary_Gentlemen_Vol_1
