# Supplementary material for: The league of extraordinary generalists: a qualitative study of professional identity and perceptions of role of GPs working on a national after hours helpline in Australia
Source: BMC Health Serv Res. 2016 Apr 22;16:142. doi: 10.1186/s12913-016-1387-5 (PMC4840489; doi:10.1186/s12913-016-1387-5)
Supplement: Additional file 1: — The interview schedule used to guide discussion with participants has been supplied. (DOCX 98 kb) [file 12913_2016_1387_MOESM1_ESM.docx]

**Evaluation of the After Hours GP helpline: GP Provider Experience Study**

**Provider In-depth Interview schedule**

1. What is your role on the *after hours GP helpline*?
2. How long have you worked on the helpline?
3. What were you doing professionally before you started work on the helpline?
4. How well prepared do you think you were for taking on your role on the helpline?

Cues: Adequacy of induction; training; availability of electronic clinical decision-support resources

1. What do you like about working on the helpline?

Cues: Professional and personal benefits

1. What are the challenges or difficulties you encounter working on the helpline?
2. What degree of contact do you have with the community-based regular GPs of callers to the helpline?
   1. What is your perception of community-based GPs view of the after hours GP helpline?
3. To what extent does the after hours GP helpline add value to the health system?

Note to interviewer: Allow respondent to speak freely about perceived value but if needed: Cues: views on benefits to community re access to medical advice, benefits to community-based GPs; benefits to acute care system; health system savings; cost to system of additional service

1. How well do you think the transfer from nurse triage to GP works?

Note to interviewer: Allow respondent to speak freely about the strengths and weaknesses of the transfer process

1. Do you have any suggestions for improving the nurse to GP transfer process?
2. To what extent does the addition of the GP on the helpline add value to the nurse triage and advice process available through nurse telephone triage *services*?

Note to interviewer: Allow respondent to speak freely about perceived value

1. How do you rate your professional satisfaction in working on the after hours GP helpline?

Note to interviewer: Allow respondent to speak freely about perceived professional satisfaction/dissatisfaction

1. Do you have any suggestions for improving the after hours GP helpline?

Note to interviewer: If yes, record suggestions

1. Do you have any other comments you’d like to make regarding your experience of working on the after hours GP helpline?

Thank you for your help today – the interview is finished now
